# Magnetic Induction Phase Difference for Cerebral Hemorrhage Detection

**DOI:** 10.3390/s25010157

**Published:** 2024-12-30

**Authors:** Jie Liu, Lian Yan, Huangsen Deng, Mingxin Qin, Mingsheng Chen

**Affiliations:** 1 Department of Biomedical Engineering, Army Medical University, The Third Military Medical University, Chongqing 400038, China; jieliures@163.com (J.L.); chris_324@163.com (H.D.); qmingxin@tmmu.edu.cn (M.Q.); 2 Department of Medical Engineering, Dongnan Hospital of Xiamen University, School of Medicine, Xiamen University, Xiamen 363000, China

**Keywords:** cerebral hemorrhage, zero-flow sensor, magnetic induction phase shift

## Abstract

Magnetic induction phase shift is a promising technology for the detection of cerebral hemorrhage, owing to its nonradioactive, noninvasive, and real-time detection properties. To enhance the detection sensitivity and linearity, a zero-flow sensor was proposed. The uniform primary magnetic field and its counteraction were achieved. Phase-change responses to solutions of varying conductivities and rabbits with cerebral hemorrhage were investigated and compared with traditional sensors. The sensitivities in detecting solutions with different conductivities were 1.84, 1.39, and 1.22 times higher than those for a low-pass birdcage coil, planar gradiometer, and Bx-sensor, respectively. The results for rabbits with cerebral hemorrhage showed that the sensitivities increased by 1.17, 1.67, and 6.3 times compared with a low-pass birdcage coil, symmetric cancelation-type sensor, and single co-axial coil, respectively. This sensor could accurately detect three stages in the pathological process. Blood loss of 1 mL meant that the compensatory mechanism of cerebrospinal fluid began to fail, and 1.4 mL of blood loss meant that the compensatory mechanism failed completely. The adjusted R-squared value of the first-order linear fit was above 0.98 in both physical and animal experiments, indicating that high detection linearity was achieved. The proposed sensor provides a more accurate method for cerebral hemorrhage detection and facilitates the practical application of magnetic induction phase shift in pre-hospital and bedside real-time detection.

## 1. Introduction

Cerebral hemorrhage (CH), characterized by non-traumatic cerebral parenchymal vascular rupture, is a type of stroke with high rates of morbidity, disability, and mortality, as well as a poor prognosis. In addition, it poses a huge threat to the life and health of the public [1,2], and its mortality ranges from 30% to 40% in the early stage, with no trend of improvement [3]. Population aging is one of the potential factors for the continued increase [4], along with low income levels [5]. Thus, early detection and real-time bedside monitoring are extremely important for improving the treatment and prognosis of patients. Currently, computed tomography (CT) is the gold standard for detecting CH because of its advantages of precise quantification and localization. However, CT has disadvantages, such as tissue or cell damage caused by ionizing radiation, along with high cost. Real-time monitoring and pre-hospital detection cannot be achieved using CT. Although the emergence of mobile CT has solved the problem of bedside detection to a certain extent, it is not always available. In addition, continuous and real-time bedside detection is of immense significance for conservatively managed and postoperative patients, especially those in the intensive care unit (ICU).

Magnetic induction phase shift (MIPS) is based on the theory of electromagnetic fields and the electrical characteristics of biological tissues, with the advantages of non-contact, noninvasive, and real-time continuous detection [6,7]. Furthermore, the low conductivity of the skull does not affect the propagation of the magnetic field [8,9]. Therefore, MIPS can achieve the initial diagnosis in the pre-hospital stage, along with long-term and continuous bedside monitoring of the lesion development. Peter P. Tarjan et al. [8] used magnetic induction detection techniques for biological measurements for the first time in 1968. Griffiths et al. [10] designed a single-channel MIT system for imaging normal saline, and the results showed that the imaginary part of the induced field was related to the conductivity. Scharfetter et al. [11] proposed magnetic induction spectroscopy (MIS) to measure the passive electronic properties of biological tissues. The results showed that four important error sources, including conductor movement, thermal drifts, lateral displacement of the excitation coil, and phase shift in the detection coil, had much less effect on electrical conductivity than dielectric permittivity and magnetic permeability [11]. Waston et al. [12] proposed a primary field compensation scheme by aligning the receiver coils along the magnetic streamlines of the primary excitation field such that no net flux cut through the coil, which was named zero flow by Scharfetter et al. [13], and this group designed a new zero-flow gradiometer (ZFGRAD) for magnetic induction tomography (MIT). Gonzalez et al. [14] designed the co-axial coil and tested the functional relation of phase difference and normal saline volume using a spherical model; the results found a positive relationship among phase difference, frequency, and volume. In the same year, this group conducted an abdominal edema detection experiment in an animal model, and the results were consistent with the preliminary theoretical research and physical testing [15]. Gonzalez et al. [16] detected rats with brain ischemia by inductive phase shift, and they proposed that the phase difference was related to the ischemic time. In 2013, this group found that the phase difference could distinguish patients with cerebral edema and cerebral hemorrhage when the excitation frequency was set at 153–166 MHz [17]. Jin et al. [18] designed a symmetric cancelation-type sensor, which considered the natural symmetry of the left and right hemispheres of the brain. They used two receiving coils to receive signals produced by the injured and normal hemispheres. The phase difference generated only by the bleeding sites was obtained by subtracting the result from the two hemispheres. The result from rabbits with CH demonstrated that the sensitivity of this sensor was higher than that of the co-axial coil [18]. Xiao et al. [19] proposed a cambered coil array and analyzed three-dimensional (3D) image performance with the brain model. The results showed that cambered MIT had higher sensitivity near the sensors than cylindrical sensor arrays [19]. The Bx-sensor [20], which utilizes the orthogonal relationship between the detection and excitation coils, is a kind of sensor structure that alleviates the effect of the primary field and improves the detection sensitivity. Imaging quality and distinguishing ability for hemorrhages with different deviation positions, depths, and volumes were analyzed using single and combined Bx-sensors [20]. The results showed that the combined structure had better image performance. The gradiometer comprises two symmetric receiving coils and one excitation coil. It is a typical sensor structure that reduces the effect of the main magnetic field and increases sensitivity. Chen et al. [21] proposed a planar sensor array with gradiometers, which had higher detection accuracy. The numerical simulation results indicated that this sensor structure could effectively alleviate the influence of background signals generated by the normal tissue of the brain. S. Haikka et al. [22] compared the sensitivities between helmet coil and circular arrays [23]. The results showed that helmet coil arrays augmented the sensitivity by enabling the placement of additional coils near the surface of the head, compared with annular coil arrays. Nonetheless, the excitation coil of the symmetric cancellation coil and co-axial coil, and even the Bx-sensor and gradiometer, is solenoid, the primary field is non-uniform and divergent, and the direction of the magnetic field vector in the detection region is inconsistent; as a result, the detection linearity can be improved. In addition, because of the weak conductivity of the biological tissues, the magnetic field strength needs to be further increased by adjusting the energy transmission of the ports to enhance the detection signal. Moreover, only numerical and physical simulations have been conducted in research on both Bx-sensors [20] and gradiometers [21]; with no experiments on animals with CH, the detection performance of these two sensors in vivo has been unclear.

This paper proposes a zero-flow sensor with primary magnetic field counteraction and higher field strength to improve detection sensitivity and linearity. A uniformly distributed main magnetic field was generated in the center of the area, which increased the detection signal and partially diminished the effect of locating the hematoma. The adjusted R^2^ value of the first-order linear fitting was 0.98, and the linearity was better than that of traditional sensors in the physical simulation experiment. Finally, rabbits with CH were detected, and the bleeding volume in different pathological stages was accurately measured. The cerebrospinal fluid (CSF) compensation mechanism began to fail when the bleeding volume was 1 mL. When the bleeding volume reached 1.4 mL, no CSF was present in the cranial cavity, indicating that the compensation mechanism totally failed and intracranial pressure would increase sharply.

## 2. Materials and Methods

### 2.1. Detection Principle

Under normal physiological conditions, the components of the brain, including cerebral blood flow (CBF), CSF, white matter, and gray matter, are maintained in a dynamic balance, and the overall conductivity rarely changes. Hence, the phase difference is theoretically consistent. However, when CH occurs, this balance is disrupted, which leads to alterations in the conductivity of the entire brain. In such instances, the phase difference reflects the changes over time. The transmitting coil, excited by a sinusoidal signal, generates the primary magnetic field (B). Subsequently, an eddy current containing the conditions and information of the object is induced by this primary magnetic field (B) and forms the secondary magnetic field (ΔB) over the target region. The receiving coil detects the superimposed magnetic field (B + ΔB) and transmits this signal to the data processing module. This module calculates the phase difference (Δθ) between the reference and detection signals.

(1) $$\Delta \theta =\theta_{det}-\theta_{ref}$$

where θ_det_ denotes the phase of the detection signal from the receiving coil, and θ_ref_ signifies the phase of the reference signal, which is equal to the phase of the excitation signal. According to Griffiths et al. [10] and Barai et al. [24], the phase difference (Δθ) can be determined based on conductivity and frequency. If the frequency of the excitation signal remains time-invariant, the phase difference is related to the conductivity variation in the detection region of the brain.

### 2.2. Design of the Coil Model

**Figure 1:**
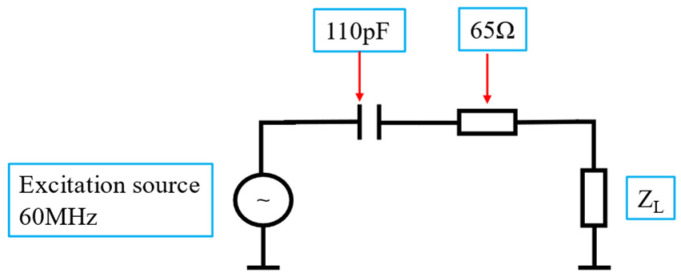
The circuit diagram of the RFEFC.

The sensor model comprised the excitation and detection coils. The excitation coil was composed of end-rings (ERs), legs, and capacitors inserted into the ERs between conductive legs. The radius of the ERs was 80 mm, and their width was 5 mm. The number of legs was set as eight, the length was 80 mm, and the width was 5 mm. According to the study of the static magnetic field, when the radii of two coils are equivalent to the distance between them, these two coils fulfill the Helmholtz condition, which makes the magnetic field the most uniform in the central region [25]. Sixteen high-frequency, high-Q-factor capacitances (TA101G, TEMEX, France) (TA101G, TEMEX, Bordeaux, France) were inserted into the ERs at equal distances. The theoretical capacitor value calculated using the BirdcageBuilder [26] at a 60.00 MHz resonance frequency was 93.63 pF. Two excitation ports were used, and the phase difference between them was 90° to achieve the orthogonal condition; thus, the signal-to-noise ratio was $\sqrt{2}$ times higher than that of the linearly polarized radio frequency coil (one excitation port) [27]. Then, the ANSYS Electromagnetics Suite (ZMT Zurich MedTech AG, PA, USA) was used to analyze the magnetic field of the sensor with different capacitors. The power of the excitation source was set as 1 W, so that the final capacitor value was selected according to the strength of the primary field and the uniformity of magnetic field distribution. Finally, the impedance was matched using a network analyzer (E5061B, KEYSIGHT, Santa Rosa, CA, USA) to achieve tuning when the S11 of two excitation ports (impedance = 50 Ω) was less than −10 dB. Here, radio frequency electronic feedback circuits (RFEFCs) were constructed to maximize the port energy transfer; the circuit diagram is shown in Figure 1. The final key parameters are shown in Table 1. The detection coil with an inner diameter of approximately 60 mm was wound using 10 turns of copper wire (diameter 1 mm). The ERs, legs, and copper wire were fixed to a 3D-printed mold made of a photosensitive resin material.

### 2.3. Experimental System

**Figure 2:**
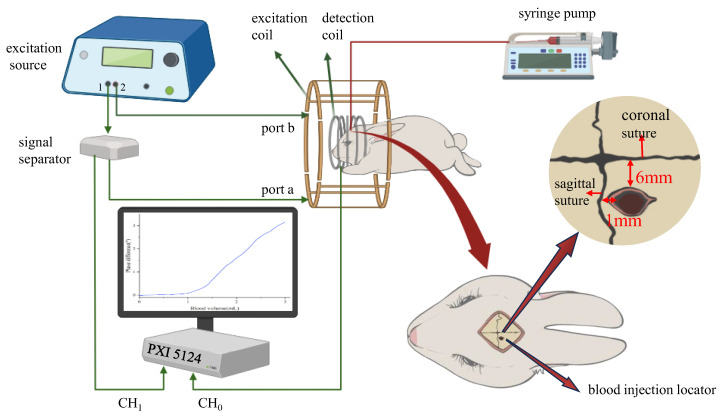
The schematic diagram of the experimental system.

The schematic diagram of the entire experimental system is presented in Figure 2. The function signal generator (AFG3252, Tektronix, Beaverton, OR, USA) generated two orthogonal sinusoidal signals of equal amplitudes (peak to peak, 5.0 Vpp) and phase difference (90 degrees) at the same frequency (60 MHz). The signal from signal generator channel port 1 (Out1) was divided into two similar signals; one of them was inputted into port “a” of the sensor as the excitation signal (V1 = 5.0 Vpp, phase = 0°), and the other was inputted into a channel (CH1) of the NI high-speed data acquisition card (PXI5124, NI, Austin, TX, USA) as the reference signal (Vref = 5.0 Vpp, phase = 0°). Another signal from signal generator channel port 2 (Out2) was inputted into port “b” as the excitation signal (V2 = V1 = 5.0 Vpp, phase = 90°). The geometric positions of ports “a” and “b” of the sensor differed by 90° to satisfy the orthogonal condition. Furthermore, the detection signal was inputted into another channel (CH0) of the NI high-speed data acquisition card (PXI5124, NI, USA). The self-programmed data processing module in LabVIEW was used to perform FFT transformation and phase difference calculation for the reference and detection signals.

### 2.4. Experimental Design

In this part, two types of liquid with different conductivities were used to simulate the overall conductivity of the brain at different stages for the same patient with CH, and to simulate the overall conductivity of the brain between different patients. Volumetric changes in a liquid with a given conductivity or different types of liquid with the same volume but varying conductivities would cause alterations in the eddy current, leading to variations in phase difference. Here, 10 mL of the artery blood of rabbits and a 0.9% NaCl solution were used. Each type of liquid was injected into a 10 mL beaker using a syringe pump (LSP01-1A, Baoding Longer, Baoding, China) at a 2 mL/min rate. Average data were obtained by repeating each experimental protocol 20 times.

Fifteen male New Zealand white rabbits (2.0–2.5 kg) with a head circumference of 17.5–18.0 cm were used in this study. Firstly, urethane (mass fraction = 25%, 5 mL/kg) was injected into the ear vein to anesthetize the rabbits. Subsequently, 3 mL of fresh autologous blood was drawn from the ear artery using a vacutainer. The local hair of the calvaria was trimmed, the scalp was cut open, and the skull cross-suture was exposed. A hole of 6 mm on the right side of the coronal suture and 1 mm behind the sagittal suture was drilled, as shown in Figure 2. A drainage tube made of optical fiber was inserted 13 mm into the skull for blood injection to achieve internal capsular hemorrhage, which is the most common form of CH. The gap between the drainage tube and the skull hole was closed using dental cement and glued to prevent the injected blood from oozing out. Before blood injection, each rabbit was detected for 10 min as the control group, and then 3 mL of artery blood was injected using a syringe pump and detected for 10 min. Real-time phase-difference data were collected using the PXI5124 card and processed via the LabVIEW program. All rabbits were alive at the end of the experiment. Finally, they were euthanatized by injecting air into their ear veins. During the experiment, the ambient temperature was controlled at 25 °C.

### 2.5. Magnetic Resonance Imaging (MRI) Analysis

MRI images of five male New Zealand white rabbits with a similar head circumference were obtained before and after injecting 1.4 mL of blood. A 3.0 T MRI device (Magnetom Spectra with A Tim + Dot System, Siemens, Munich, Germany) was utilized, an extremity knee coil was placed on the rabbit’s head, and the procedure was conducted at Southwest Hospital, Chongqing, China. T2-weighted 3D variable-flip-angle TSE (SPACE) sequences were used to clearly discriminate CSF from the surrounding tissues. The scanning parameters were set as follows: TR = 1300 ms, ETL = 49, TE = 44 ms, matrix = 320 × 275, FOV = 160 mm × 160 mm, number of slices = 192, slice thickness = 0.5 mm, and slice spacing = 0 mm. The BeeDicomViewer software (China) Vision3.6.2 (Beijing, China) was used for image processing.

### 2.6. Ethics Statement

All animal experiments were performed in accordance with the guidelines of the Administration of Animal Experiments for Medical Research Purposes issued by the Ministry of Health of China. The experimental protocol was approved by the Animal Experiments and Ethical Committee of Army Medical University (AMU, Chongqing, China). All efforts were taken to minimize the suffering of the rabbits during the experimental process.

## 3. Results

**Figure 3:**
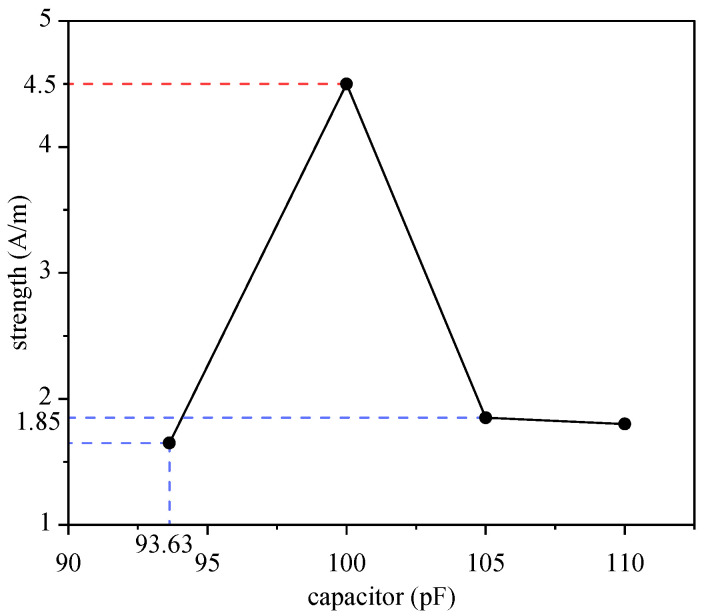
The magnetic field strength of the sensor with different capacitors.

**Figure 4:**
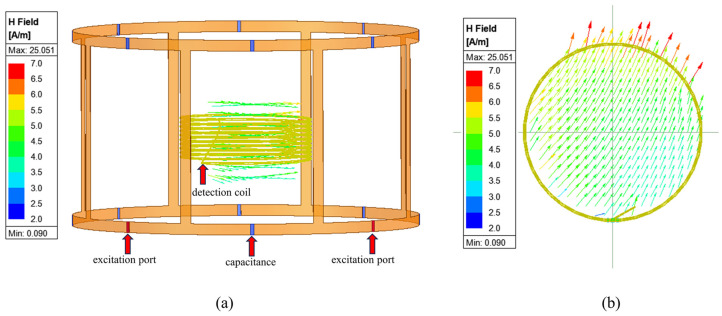
Magnetic field distribution: (**a**) The front view of the magnetic field. (**b**) The top view of the magnetic distribution.

The magnetic field strength of the sensor with different capacitors was as depicted in Figure 3. The magnetic field strength of the sensor with 100 pF reached the maximum (4.5 A/m), and the intensity decreased significantly when the capacitance was above or below 100 pF. More importantly, the angle between the main magnetic field and the detection coil was approximately 0°, as shown in Figure 4a. Figure 4b shows that a uniformly distributed magnetic field was induced instead of the dispersed magnetic field generated by the co-axial coil. The comparison of magnetic field intensity between this novel sensor and traditional sensors in the simulation experiment is shown in Table 2, where the multiple is the field strength ratio between the zero-flow sensor and the other sensors shown in the table.

**Figure 5:**
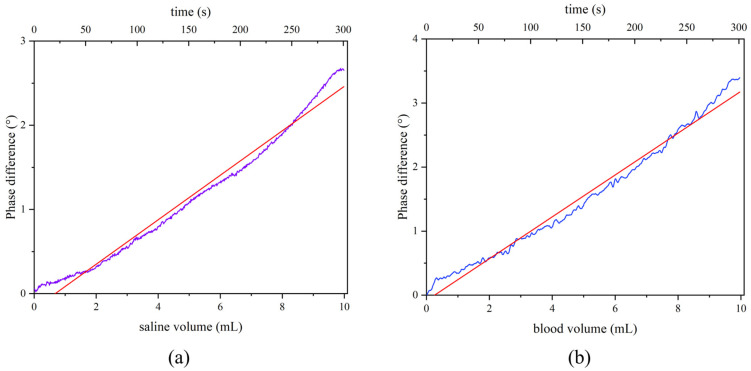
Original data of the physical simulation experiment: (**a**) 0.9% NaCl; (**b**) rabbit artery blood. The red curve was a linear fit. The blue curve was original data.

The phase difference was determined, and the average of 20 times was calculated for the 0.9% NaCl solution and the artery blood of rabbits. The original data were as portrayed in Figure 5, where a linear increasing trend with increasing injection volume was observed in both types of solution. The phase difference (mean ± standard deviation) was as shown in Table 3, and the results of the first-order linear fit with no weighting between phase difference and volume were as presented in Table 4. The comparison of sensitivity between this proposed sensor and traditional sensors in physical experiments is shown in Table 5, where the sensitivity multiplier is the phase difference of the zero-flow sensor divided by that of traditional sensors for the same bleeding volume.

**Figure 6:**
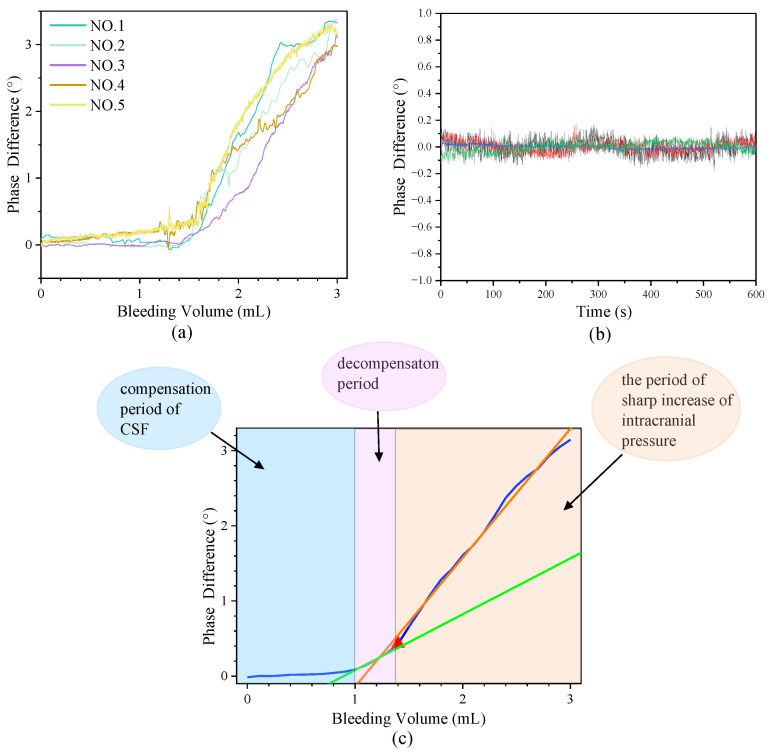
The results of the rabbit experiments: (**a**) Original data from the CH group. (**b**) Original data from the control group. (**c**) The curve of the scattered average value of the CH group.

All 15 rabbits survived after the experiment, and the results of 5 rabbits are depicted in Figure 6. The findings were similar for the other rabbits. As shown in Figure 6c, the phase difference changed slightly during the injection of the first 1 mL. Then, it increased with the blood injection volume, as shown by the green fitting line. However, after the injection of 1.4 mL (red triangle in Figure 6c), a sharp change was observed in the phase difference, as shown by the orange fitting line. The results of the first-order linear fit with no weighting are listed in Table 6. The phase difference (mean ± standard deviation) for 3 mL of artery blood was 3.15° ± 0.15°. In the control group, the phase shift remained almost unchanged during the detection stage. Table 7 compares the in vivo sensitivity of this proposed sensor and traditional sensors.

**Figure 7:**
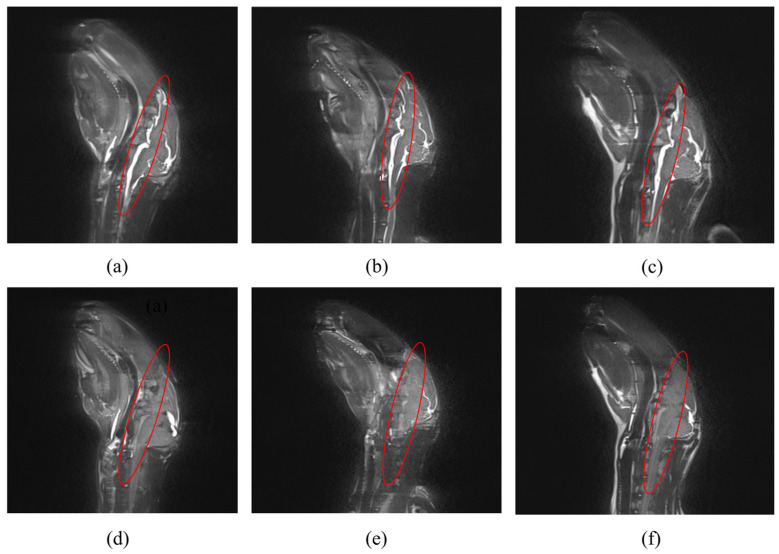
MRI of the CH group: The part inside the red ellipse indicates the CSF. (**a**–**c**) The MRI images before blood injection. (**d**–**f**) The MRI images after injecting 1.4 mL of blood.

The MRI analysis of three rabbits in the blood injection group is illustrated in Figure 7 (slice: NO_98), and the other two rabbits exhibited similar results. Figure 7a–c portray the MRI images of three rabbits before blood injection. In the figures, CSF is visible, represented by the highlighted part inside the red ellipse. However, after the injection of 1.4 mL of blood, the CSF was emptied from the cranium, as shown in Figure 7d–f.

## 4. Discussion

In this study, a magnetic induction phase difference sensor was designed for CH detection. As shown in Figure 3, the magnetic field strength of the sensor with 100 pF was 4.5 A/m. This was 2.6 times that of a low-pass birdcage coil [28] and one order of magnitude higher than that of co-axial single-transmitter–receiver coils and dual-excitation coils under similar experimental conditions. A higher magnetic field strength means higher magnetization, higher induced current in the detection coil, a stronger detection signal, and higher detection sensitivity. Furthermore, Figure 4a shows that the main magnetic field was parallel to the detection coil. As a result, the net magnetic flux of the detection coil was close to zero. Therefore, this was called the zero-flow sensor, thus maximally eliminating the influence of the main magnetic field, which is conducive to enhancing the detection sensitivity. Figure 4b shows that a uniformly distributed magnetic field was induced; as a result, the detection linearity was better than that of the co-axial sensor.

The results for liquids with different conductivities are shown in Figure 5. As the liquid volume increased, the phase difference gradually increased monotonically and linearly. The mean phase difference obtained by He et al. [28] using a low-pass birdcage coil to detect 10 mL of the 0.9% NaCl solution was approximately 1.3°. Chen et al. [21] used a planar sensor array with gradiometers to collect 12.5 mL of porcine blood with a maximum phase difference of 4°. Conversely, this study achieved a maximum value of 3.90° using only 10 mL of autologous rabbit blood, representing a 2.01-fold and 1.22-fold increase in sensitivity, respectively, compared with the previous two sensors. Furthermore, a 5 Vpp excitation voltage was used instead of the 20 Vpp excitation voltage used in planar arrays with gradiometers [21]. This finding indicates that the zero-flow sensor has greater resolution, better performance, and lower cost. The magnetic-induced tomography sensor proposed by Chen et al. [21] could image the approximate location of the hematoma in a head model phantom with limited depth. However, for patients with CH, especially conservatively managed patients and postoperative patients, the bleeding volume and location are already known from CT images at the time of their hospitalization and examination. Hence, bedside monitoring with high sensitivity is more important for these patients. Furthermore, according to Table 4, the adjusted R^2^ of the first-order linear fitting was 0.98 for both 0.9% NaCl and rabbit blood. This indicates higher linearity between phase difference and the injection volume of solutions compared to the combined Bx-sensor [20] with the same depth, due to the uniformly distributed magnetic field. An approximate positive proportional relationship was observed between phase difference and injection volume from the fitting curve trend in Figure 5. This experiment revealed the relationship among solution injection volume, changes in overall conductivity, and phase difference, thereby serving as a reference for subsequent animal experiments.

The original data of the CH group and control group of rabbits are depicted in Figure 6a,b with different colors, which are marked as NO.1–NO.5. When the same inner capsule hemorrhage model was constructed and 3 mL of artery blood was injected, the mean phase difference caused by the low-pass birdcage coil, symmetric cancelation-type sensor, and single co-axial coil was 2.709° [29], 1.885° [18], and 0.50° [30], respectively. Thus, the sensitivity of our new sensor was 1.17, 1.67, and 6.3 times higher than that of the sensors mentioned above, respectively.

Three stages in the slope of the increasing mean value of the fitting curve shown in Figure 6c could be attributed to the complex process of CH. In the first stage (bleeding volume: 0–1 mL), the CSF compensation mechanism began to work to maintain a constant intracranial pressure (ICP) and the normal functioning of the brain. Thus, the overall conductivity change was weak, and the phase difference changed only slightly, which was the compensation period of CSF. When the injection blood volume was 1 mL, the CSF content was at a very low level [31]. Subsequently, as the blood volume increased, progressive failure of CSF compensation mechanisms led to an overall increase in conductivity, and the phase difference changed. This phenomenon indicates that the self-regulation mechanism was breaking down, and the condition of the patients worsened in the second stage. More importantly, in the third stage, after the bleeding volume reached 1.4 mL (red triangle in Figure 6c), the phase difference increased drastically with the increasing blood injection volume. This indicates that the CSF compensation mechanism totally lost its function, and the ICP began to rise sharply. In other words, the CSF volume of the brain was 1.4 mL. This result was our new finding in magnetic phase difference for CH detection. The MRI analysis depicted in Figure 7 showed that when the bleeding volume reached 1.4 mL, almost no CSF was present in the cranial cavity. This phenomenon, to a certain extent, supports our findings in this study. Figure 6 also demonstrates that this system was more suitable for clinical monitoring scenarios, especially for patients in the ICU and conservatively treated patients. This is because, in clinical practice, changes in the physiological condition of patients are of greater significance for doctors and nurses than the bleeding volume alone, since the tolerance or adjustment ability of patients with CH varies. The clinician can judge the pathological status of the patient according to the different stages of the phase-difference curve. Differences in the compensation abilities of each experimental rabbit may lead to variances in the slopes of the curves, but the trend was similar. The adjusted R^2^ in the first stage (0–1 mL) was 0.89, as shown in Table 6; the linearity was not very good, because the overall conductivity of the brain changed slightly, and the phase difference might have been influenced by environmental electromagnetic interference more easily. In the second and third stages, the adjusted R^2^ was above 0.98. This means that the detection linearity was better in these two stages, and these results were in accordance with the theoretical analysis and physical experiments.

## 5. Conclusions

The findings emphasized that a uniform magnetic field with higher field strength and primary field cancelation was achieved. This sensor could detect the phase difference induced by increased hemorrhage volume more accurately. More importantly, it enabled the quantitative detection of a series of brain-content diseases. Furthermore, it effectively signified the failure of the CSF compensation mechanism, indicating the possible increase in ICP and providing a novel method to monitor the condition of ICU patients or conservatively treated patients. However, its sensitivity in the early stages of hemorrhage and its anti-interference need to be improved. An electromagnetic shielding module for this sensor could be designed to improve its anti-interference. A 3D-printed coil headgear or flexible coil and wireless data transmission to intelligent terminals could be employed to make it portable. ICP monitoring could be utilized to identify the association between phase difference and ICP. In the future, an excitation source with higher power and new materials with better electrical conductivity will be used to enhance its sensitivity in the early stages.

## Figures and Tables

**Table 1:** Key parameters of the excitation coil.

Parameter	Value
Frequency	60 MHz
Length	90 mm
Radius	80 mm
Thickness	0.1 mm
Capacitor	100 pF

**Table 2:** The comparison of magnetic field intensity.

Sensor	Intensity (A/m)	Multiple
Low-pass birdcage	1.725	2.6
Co-axial	0.15	30

**Table 3:** The mean value and standard deviation of the phase difference.

	0.9% NaCl Solution	Rabbit Blood
Mean	2.37°	3.34°
Standard deviation	0.09°	0.22°

**Table 4:** The results of the linear fit.

	0.9% NaCl	Rabbit Blood
Fitting function	y = a × x + b	
a	0.26421 ± 0.00132	0.32683 ± 0.00148
Adjusted R^2^	0.98167	0.98484

**Table 5:** The sensitivity multiplier in physical experiments.

Sensors	Phase Difference	Sensitivity Multiplier
Birdcage coil	1.5°	1.84
Bx-sensor	1.7°	1.39
Planar gradiometer	3.2°	1.22

**Table 6:** The fitting results of the CH group.

Bleeding Volume	0–1 mL	1–1.4 mL	1.4–3 mL
Fitting function	y = a × x + b		
a	0.0829 ± 0.0091	0.7439 ± 0.04336	1.7153 ± 0.03557
Adjusted R^2^	0.8912	0.98988	0.99316

**Table 7:** The sensitivity multiplier in animal experiments.

Sensor	Phase Difference	Sensitivity Multiplier
Birdcage coil	2.709°	1.17
Symmetric cancelation coil	1.88°	1.67
Single co-axial coil	0.5°	6.3

## Data Availability

Data are contained within the article.
